# *Ganoderma*: A Cancer Immunotherapy Review

**DOI:** 10.3389/fphar.2018.01217

**Published:** 2018-10-25

**Authors:** Yu Cao, Xiaowei Xu, Shujing Liu, Linfang Huang, Jian Gu

**Affiliations:** ^1^Institute of Medicinal Plant Development, Chinese Academy of Medical Sciences & Peking Union Medical College, Beijing, China; ^2^Department of Pharmacy, Southwest University for Nationalities, Chengdu, China; ^3^Department of Pathology and Laboratory Medicine, Perelman School of Medicine, University of Pennsylvania, Philadelphia, PA, United States

**Keywords:** *Ganoderma*, lingzhi, bibliometrics, cancer immunotherapy, mechanism

## Abstract

*Ganoderma* is a significant source of natural fungal medicines and has been used for the treatment of various diseases for many years. However, the use of *Ganoderma* in cancer immunotherapy is poorly elucidated. In this study, we have analyzed 2,398 English-language papers and 6,968 Chinese-language papers published between 1987 and 2017 by using bibliometrics. A steady growth in the number of publications was observed before 2004, followed by an exponential increase between 2004 and 2017. The most common category for publications about *Ganoderma* was “Pharmacology & Pharmacy,” in which immunomodulation (25.60%) and cancer treatment (21.40%) were the most popular subcategories. Moreover, we have provided an overview of the bioactive components and combinatorial immunomodulatory effects for the use of *Ganoderma* in the treatment of cancer, including the major pathways of immune cells. Immunomodulatory protein and polysaccharides are the key bioactive factors responsible for cancer immunotherapy, and the NF-κB and MAPK pathways are the most comprehensively investigated major pathways. Our results indicate that *Ganoderma* has a broad-spectrum application for the treatment of cancer through the regulation of the immune system. This review provides guidance for future research into the role of *Ganoderma* in cancer immunotherapy.

## Introduction

*Ganoderma*, also called Lingzhi, is one of the most well-known medicinal species. Regarded as the “marvelous herb,” it is used widely in China, America, Japan, Korea, and other countries (Meng et al., 2011). According to traditional Chinese medicine (TCM) theory, *Ganoderma* has the ability to enhance body resistance, i.e., “Fuzheng Guben” (Yue et al., 2006). “Channel tropism” (Gui-Jing) links the functions of herbal drugs to their corresponding internal organs, channels, and various body parts to allow the interpretation of their functional mechanisms. The channel tropism of *Ganoderma* is the heart, lung, and liver, according to Gui-Jing theory. The main *Ganoderma* species are *G. lucidum, G. sinensis, G. applanatum, G. tsugae, G. atrum*, and *G. formosanum*. *G. lucidum* and *G. sinensis* are recorded in ChP2015 (Pharmacopeia of the People's Republic of China), and *G. lucidum* is recorded in USP40-NF35 (U.S. Pharmacopeia/National Formulary; Gao et al., 2004). The production of *Ganoderma* occurs mainly through artificial cultivation, which has provided an abundance of materials for the market; the yield has already surpassed that of wild *Ganoderma* (Chen et al., 2017). Methods used for *Ganoderma* identification include microscopy, TLC, spectroscopy, chromatography, chemical fingerprinting, and DNA sequencing. DNA sequencing has recently been used for classification of different *Ganoderma* species, with HPLC, UPLC, LC-Q-TOF-MS, HPTLC, and GC-MS have been commonly applied for quality evaluation (Toh Choon et al., 2012; Hennicke et al., 2016). *Ganoderma* has been used for the clinical treatment of chronic bronchitis, bronchial asthma, leukopenia, coronary heart disease, arrhythmia, and acute infectious hepatitis. However, at present, it does not have the potential to be used as first-line therapy, but only as an addition to conventional therapy in a clinical setting (Gao and Zhou, 2003; Unlu et al., 2016).

Chemical drugs for cancer treatment, such as cisplatin and cyclophosphamide, can cause side effects, such as nephrotoxicity, which are detrimental to the quality of life of patients (Aguirre-Moreno et al., 2013). In addition to this toxicity, the resistance of some cancer cells to treatment has led to the need for the evaluation of alternative approaches. Hence, chemotherapy does not completely meet the treatment need and immunotherapy is a promising alternative method as it results in fewer side effects. The use of cancer immunotherapy has gained acceptance because immune cells play notable roles in the control of cancer (Blattman and Greenberg, 2004). Immune cells can identify cancer cells as dangerous and consequently attack them; thus, the use of cancer vaccines to treat growing tumors is considered an excellent therapeutic strategy (Rosenberg et al., 2004). Herbal medicines have also been examined in clinical trials for cancer immunotherapy. Shing et al. found that a 6 months treatment using *G. lucidum* increased the mitogen-induced lympho-proliferative responses in immunocompromised children with tumors (Shing et al., 2008).

Bibliometrics is a method of document analysis that can count and analyze a large number of articles and monitor the trends in research (Kim and Park, 2011). Previous studies have reviewed the anticancer and/or immunomodulatory effects of *G. lucidum* and their potential immunological mechanisms (Lin and Zhang, 2004; Xu et al., 2011). However, the bioactive substances and corresponding immunoregulatory effects of *Ganoderma* in the treatment of cancer have not yet been investigated. Therefore, we have provided an overview of the research trend on *Ganoderma* determined from bibliometrics and reviewed its bioactive components and combinatorial immunomodulatory effects for use as a cancer treatment. We have also summarized the major diseases and pathways involved, clinical studies, and preliminary assessments of toxicity.

## Literature analysis

Bibliometrics is defined as the application of statistics and mathematics to analyze bibliographical metadata linked to scholarly publications. Bibliometrics uses a literature system and literature metrology characteristics as research objects to quantitatively and qualitatively analyze the studies. Bibliometrics can be used to monitor the trends in the scientific development of a research domain; it can be used to analyze the trends and provide a comprehensive perspective on a topic. Therefore, we analyzed a specific question from the review of published literature by using current software programs (Aggarwal et al., 2016). Using professional bibliometrics software, such as CiteSpaceV (Chen et al., 2014) and RAWGraphs, we performed a bibliometric analysis of the publications on Ganoderma between 1987 and 2017 from the Web of Science (WoS), PubMed, and CNKI databases, which were the most suitable databases for this type of evaluation. We found 2,205 articles in WoS and 1,368 articles in PubMed with “Ganoderma,” “Lingzhi,” or “Reishi” as the key words. After removal of the duplicates, a total of 2,398 English-language articles (included in the Science Citation Index) were retrieved. We also found 6,968 Chinese-language articles on CNKI with the Chinese word for “Lingzhi” as the key word. We analyzed publication counts, cooperation between countries, and research categories. We found that immunomodulation and antitumor research were the most popular research subcategories; subsequently, from examination of the relevant literature, the topic of this review was determined to be cancer immunotherapy.

### Publication counts

**Figure 1:**
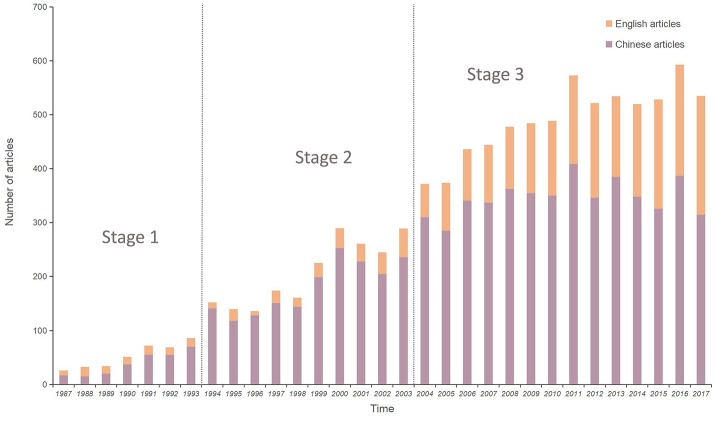
Statistical analysis for published articles of genus *Ganoderma*.

The publication counts for each year from 1987 to 2017 are shown in Figure 1. From on the number of publications, This 30 years period was preliminarily divided into three stages: Stage 1, from 1987 to 1993, was considered as the budding period, when <100 papers were published annually; Stage 2, from 1994 to 2003, was known as the development period, when the number of annual publications increased linearly from 100 to 300; Stage 3, from 2004 to 2017, was the “boom period,” when the annual number of papers increased rapidly; in particular, the number of English-language papers doubled annual. Research interest into *Ganoderma* widened over the years examined; moreover, the number of English-language studies has recently increased rapidly, revealing the potential research value of *Ganoderma*.

### Cooperation between countries

**Figure 2:**
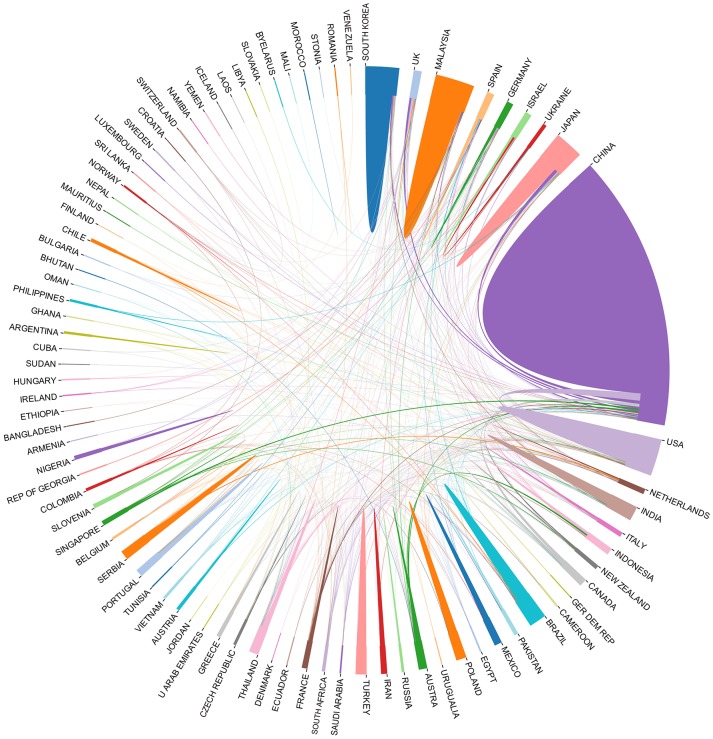
Statistical analysis for relationship among countries for *Ganoderma* research. Different countries are represented by different colors, and the size represents the number of publications.

The relationships between many countries with active *Ganoderma* researchers, based on their publications included in the Science Citation Index, are illustrated in Figure 2. In total, 84 countries were involved in the study of *Ganoderma*. China, the United States, Malaysia, Japan, and South Korea have the highest output and the most extensive cooperation was found among these countries.

### Subject categories and major historical developments

**Figure 3:**
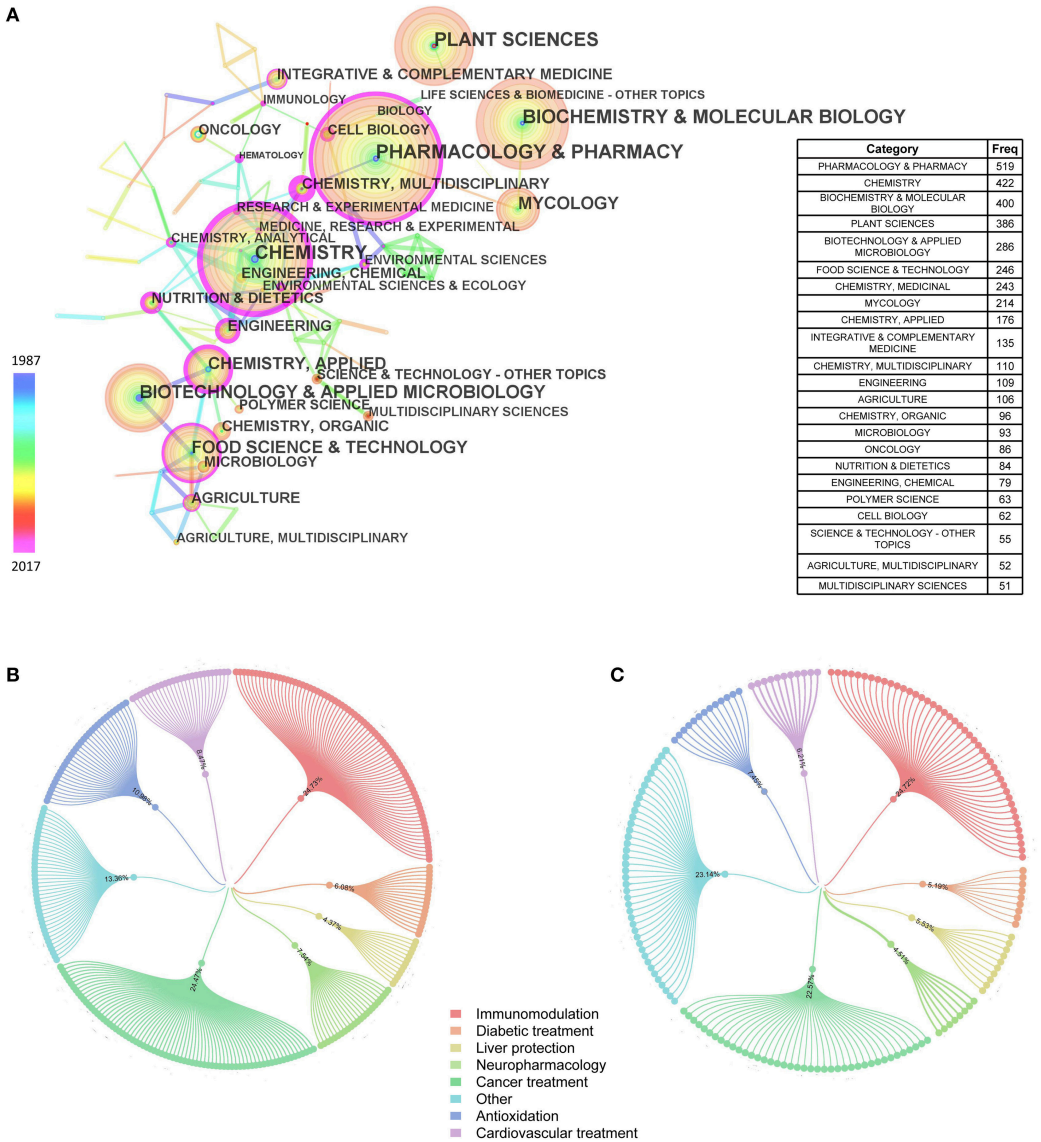
Analysis for subject categories of *Ganoderma*. **(A)** Subjects of 50 frequencies or more (included in Science Citation Index). Nodes represent objects analyzed. And the larger nodes, the more frequently they occur. The connections among nodes represent the cooperative relationships. The thicker the connections, the closer they consociate. **(B)** Classification of pharmacological effects in Chinese articles **(C)** Classification of pharmacological effects in English articles.

The categories of articles about *Ganoderma* that were included in the Science Citation Index are shown in Figure 3A. After the software analysis, we have displayed only subjects with a frequency of 50 or more. The most abundant category, “Pharmacology & Pharmacy,” had a frequency of 519, followed by the categories of “Chemistry” (422) and “Biochemistry & Molecular Biology” (400). From further reading, we found 1,512 Chinese-language articles and 880 English-language articles included in the Science Citation Index that described the pharmacological effects of *Ganoderma*. These pharmacological effects were subdivided into several specific effects (Figures 3B,C), such as immunomodulation, cancer treatment, antioxidation, cardiovascular treatment, diabetic treatment, liver protection, and neuropharmacology. The immunomodulation effect-related studies occupied the largest proportion of the eight areas of pharmacology, followed by cancer treatment, both in Chinese-language articles (24.73 and 24.47%, respectively) and in English-language articles (24.72 and 22.57%, respectively). Furthermore, in English-language articles, the number of citations was 17,692 and the average citation per item, which is the average number of articles cited for all items in the results set, was 20.43.

**Figure 4:**
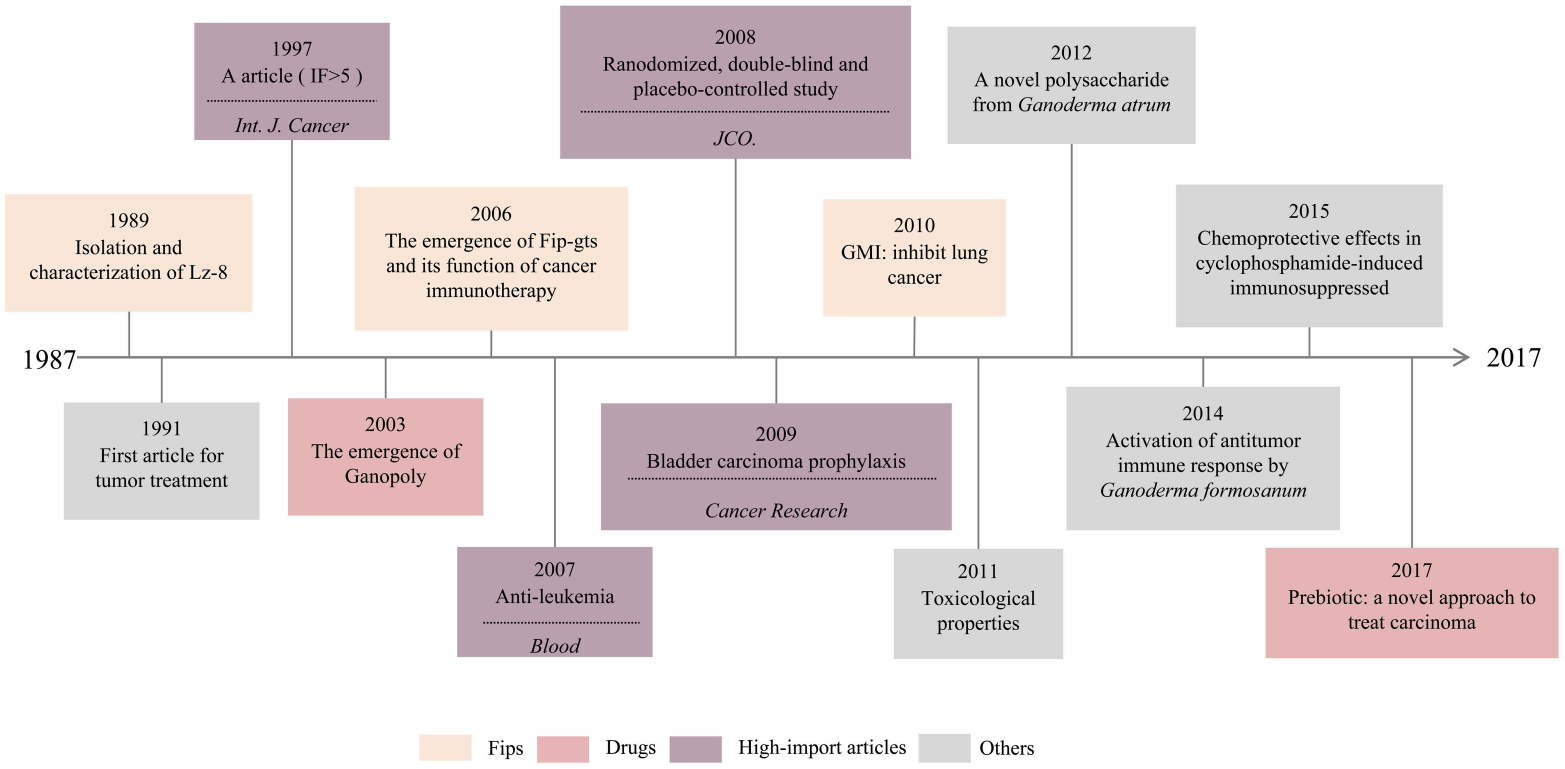
Timeline of major historical developments of *Ganoderma* on cancer treatment.

Further analysis of the English-language articles led to the identification of a total of 196 articles related to cancer immunotherapy. The timeline of major historical developments that are related to *Ganoderma* in cancer immunotherapy is shown in Figure 4. We found three types of fungal immunomodulatory proteins (Fips) that played important roles; Lz-8 was the first of these discovered. Moreover, the first study of the effect of *Ganoderma* on the inhibition of tumor growth occurred as early as 1991. In 2003, Ganopoly appeared as a new drug, and has since been used widely in clinical practice. Furthermore, the toxicology and immunology of *Ganoderma* were partly addressed in 2011 and its chemoprotective effects against cyclophosphamide-induced immunosuppression were studied in 2015. In addition, prebiotics were investigated as a novel approach for the treatment of carcinoma in 2017. Cancer immunotherapy has emerged one of the most popular fields of *Ganoderma* research. Hence, we have focused on the immunomodulatory effects of this genus and its constituent active components for use in cancer treatment.

## Immunomodulatory effects of *ganoderma* and its active components on cancer treatment

Many pharmacological and clinical studies have shown that *Ganoderma* can play an antitumor role through the regulation of the immune system (Boh et al., 2007). The therapeutic effects of *Ganoderma* are attributed to fungal immunomodulation proteins (FIPs), polysaccharides, and triterpenoids. Furthermore, we have specifically summarized active components of *Ganoderma* and their corresponding pharmacological effects.

### Fungal immunomodulation proteins

FIPs are small molecular proteins purified from various fungi, such as *Ganoderma*. These proteins are functional families of *Ganoderma* components with anticancer effects (Table 1). Four types of immunoregulatory proteins, Lingzhi-8 (Lz-8), Fip-gts, GMI, and Fip-gat, have been isolated and purified from *Ganoderma*.

**Table 1:** Pharmacological effects of immunomodulatory proteins of *Ganoderma*.

**Source**	**Protein**	**Cell lines/Mice**	**Optimum treatment concentration/dose**	**Duration**	**Pharmacological effect(s)**	**References**
*G. lucidum*	(r)Lz-8	A549, CL1-5, H226, LLC1 cells, C57BL/6 mice	5 μg/ml	12 h, 4 weeks	Induced changes in epithelial to mesenchymal transition by interfering with cell and focal adhesion kinase (FAK) functions in lung cancer cells.	Lin and Hsu, 2016
		SGC-7901 cell	0.5 μg/ml	24 h	Induced endoplasmic reticulum stress-mediated autophagic cell death.	Liang et al., 2012
		Human primary and Jurkat T cells	1 μg/ml	24 h	Induced IL-2 gene expression via the Src-family protein tyrosine kinase.	Hsu et al., 2008
		LLC1 cell, C57BL/6 mice	10 μg/ml, 7.5 mg/kg	48 h, 18 days	Inhibited growth and induced apoptosis of lung cancer cells by promoting epidermal growth factor receptor (EGFR) degradation.	Lin et al., 2017
		MBT-2 cell, C57BL/6, C3H/HeN, C3H/HeJ mice	10 μg/ml	90 days	Improved the therapeutic effect of DNA vaccine against MBT-2 tumor in mice.	Lin et al., 2011
*G. tsugae*	(r)Fip-gts	HeLa, SiHa, and Caski cells	0.15 μM	24 h	Suppressed cervical cancer cell migration and enhanced the inhibition of FIP-gts upon migration.	Wang P. H. et al., 2007
		A549, MRC-5 cells	8 μg/ml	48 h	Regulated telomerase in A549 cells.	Liao et al., 2006
		A549, H1299, A549-p53, H1299-p53 stable cells	1.2 μM	48 h	Induced suppression of telomerase activity in lung cancer cells by post-translational modifications of hTERT protein	Liao et al., 2007
		A549, CaLu-1 cells, nude mice	1.2 μM, 12.8 mg/kg	48 h, 33 days	Inhibited A549 cell growth. A549 cells treated with reFIP-gts grew slower than cells treated with PBS alone *in vivo*.	Liao et al., 2008
*G. microsporum*	GMI	A549, CaLu-1 cells, nude mice	1.2 μM, 160 μg/mouse	48 h, 66 days	Induced lung cancer cell death by activating autophagy, but did not induce apoptotic cell death.	Hsin et al., 2011
		A549, CCL-185 cells	8 μg/mg	24 h	Exhibited an inhibitory effect on EGF-induced migration and invasion.	Lin et al., 2010
		A549, CaLu-1 cells	1.2 μM	48 h	Inhibited lysosome degradation on autophagosome formation	Hsin et al., 2012
		A549, CaLu-1 cells	1.2 μM (GMI) + 5 μM (Cisplatin)	48 h	Induced apoptosis via autophagy and might be a potential cisplatin adjuvant against lung cancer.	Hsin et al., 2015
*G. atrum*	(r)Fip-gat	MDA-MB-231 cell	9.96 μg/ml	48 h	Triggered significant cell cycle arrest at the G1/S transition and pronounced increase in apoptotic cell population.	Xu et al., 2016

Lz-8, an immunomodulatory protein from *G. lucidum*, was first isolated and cloned in 1989. Primarily composed of 110 amino acids, Lz-8 has an immunoglobulin-like structure that forms non-covalently linked homodimers with biological activity (Kino et al., 1989). Lz-8 exerted significant therapeutic effects on gastric cancer and specific lung cancers. Liang et al. found that recombinant Lz-8 (rLz-8) induced autophagic cell death through aggregation in the endoplasmic reticulum (ER), which triggered ER stress and the ATF4-CHOP pathway in SGC-7901 human gastric cancer cells (Liang et al., 2012). Moreover, rLz-8 might be a useful chemotherapeutic agent for the treatment of lung cancer because owing the key role of FAK targets in metastasis (Lin and Hsu, 2016). In addition, Lin et al. reported a novel anticancer effect of rLz-8 through targeting EGFR mutation or overexpression and EGFR-dependent processes in lung cancer cells (Lin et al., 2017).

Fip-gts is an immunomodulatory protein purified from *G. tsugae*. The DNA encoding this protein was isolated from a cDNA library by using a reverse transcriptase-polymerase chain reaction (Lin et al., 1997). The recombinant FIP-gts (rFip-gts) suppressed telomerase activity in a dose-dependent manner through the downregulation of the telomerase catalytic subunit (Liao et al., 2006). RFip-gts inhibited telomerase activity in lung cancer cells *in vitro* through effects on nuclear export mechanisms, which may have been mediated by the ER stress-induced intracellular calcium level (Liao et al., 2007). *In vivo* studies showed that the growth of A549 cells in nude mice treated with rFIP-gts was significantly slower than those treated with PBS, which confirmed that lung tumor growth could be inhibited by rFIP-gts (Liao et al., 2008). Moreover, this protein was also shown to affect cervical cancer cells.

GMI is an immunomodulatory protein cloned from *G. microsporum*. The amino acid sequence of this protein shared 83% homology with that of FIP-gts (Chiu et al., 2015). *In vitro* studies found that GMI inhibited the EGF-induced phosphorylation and activation of EGFR and AKT pathway kinases in a dose-dependent manner (Lin et al., 2010). Hsin et al. found that autophagosomal accumulation induced autophagic cell death in a model of GMI treatment, and ATP6V0A1, a subunit of vesicular H^+^-ATPases, regulated autophagosome lysosome fusion. Hsin et al. also revealed that GMI and cisplatin induced apoptosis via autophagy/caspase-7-dependent and survivin- and ERCC1-independent pathways (Hsin et al., 2012). *In vivo* studies suggested that the oral administration of GMI inhibited tumor growth and induced autophagy in nude mice that were administered a subcutaneous injection of A549 cells (Hsin et al., 2011).

Fip-gat is an immunomodulatory protein from *G. atrum* containing 111 amino acids. Xu et al. treated MDA-MB-231 cells with different concentrations of recombinant Fip-gat *in vitro* and found that this protein reduced cell viability in a dose-dependent manner (Xu et al., 2016). Treatment with FIP-gat triggered a significant degree of cell cycle arrest in the G1/S transition and a pronounced increase in the apoptotic cell population.

### Polysaccharides and other active components

Polysaccharides (Meng et al., 2014) and other active components of *Ganoderma* also play key roles in its use for cancer treatment owing to their immunomodulatory effects (Table 2). Their effects are described below relative to different diseases.

**Table 2:** Pharmacological effects of other bioactive components than proteins of *Ganoderma*.

**Source**	**Components**	**Cell lines/mice**	**Optimum treatment concentration/Dose**	**Duration**	**Pharmacological effect(s)**	**References**
*G. lucidum*	Water extract	γ-ray-irradiated mice	400 mg/kg	35 days	Enhanced the recovery of cellular immuncompetence from γ-ray-irradiation.	Chen and Hau, 1995
		RAW 264.7 cell	100 μg/ml	24 h	Inhibited LPS-induced NO production in RAW 264.7 macrophages.	Song et al., 2004
		NK92, pNK, K562 cells	5% effector/target ratio	24 h	Induced NK cell cytotoxicity against various cancer cell lines by activating NKG2D/NCR receptors and MAPK signaling pathways.	Chang et al., 2014
	Ethanolic extract	MDA-MB 231, B16-F10 cells	250 μg/ml	48 h	Decreased the viability of both cancer cells in a time- and concentration-dependent manner.	Barbieri et al., 2017
	Polysaccharide	HL-60 and U937 cells	100 μg/ml	5 days	Increased IL-1 and IL-6 and might play an indirect role in potentiating anti- tumor immunity *in vitro*.	Wang et al., 1997
		C57BL/6j, BALB/c mice	12.8 mg/L	5 days	Promoted the cytotoxicity of specific cytotoxic T-lymphocytes induced by dendritic cells (DC), which were pulsed with P815 tumor antigen during the stage of antigen presentation.	Cao and Lin, 2003
		LAK cells, C57BL/6j mice	400 or 100 mg/L	8 days	Mediated the anti-tumor activity through complement receptor type 3.	Zhu and Lin, 2005
		L929, P815, YAC-1 cells, C57BL/6 mice	400 or 100 mg/L	15 days	Promoted cytokine-induced killer (CIK) cell proliferation and cytotoxicity were relevant to enhancing IL-2, TNF production.	Zhu and Lin, 2006
		S180, Heps, EAC cells, ICR species mice	300 mg/kg	8 days	Inhibited the growth of inoculated S180, Heps, and EAC tumor cells in mice.	Pang et al., 2007
		S180 cell, BALB/c mice	200 mg/kg	14 days	Activated the immune response of the host organism by the stimulation of NK cells, T cells, and macrophages.	Wang et al., 2012
		rats of Wistar strain	2.6 mg/ml	48 h	Enhanced the antioxidant enzyme activities, and reduced levels of IL-1b, IL-6, and TNF-α in rats with cervical cancer.	Chen et al., 2009
		B16F10 cell, C57BL/6 and BABL/c mice	12.8 μg/ml	72 h	Had antagonistic effects on the immunosuppression induced by B16F10 culture supernatant.	Sun et al., 2011a
		B16F10 cell, BALB/c mice	400 μg/ml	5 days	Suppressed lymphocyte proliferation and perforin and granzyme B production in lymphocytes after induction with phytohemagglutinin.	Sun et al., 2011b
		B16F10 cell	400 μg/ml	48 h, 21 days	Enhanced major histocompatibility complex (MHC) class I, more efficient immune cell-mediated cytotoxicity against these B16F10 cells might be induced.	Sun et al., 2012
		B16, A375 cells, C57Bl/6J mice	400 μg/ml	21 days	Inhibited the adhesion of fibrinogen to melanoma cells and reversed the blocking effect of the fibrin coat on NK cytotoxicity against melanoma cells.	Zheng et al., 2012
		HepG2 cell	Unknown	Unknown	Inhibited HepG2 cells directly through regulation of hepato-carcinoma genes.	Shen et al., 2014
		Lymphocytes of cancer patients	12.8 μg/ml	48 h	Antagonized lung cancer patient plasma-induced suppression of lymphocyte activation by phytohemagglutinin.	Sun et al., 2014
		H22 cell, Kunming, BALB/c male mice	200 mg/kg	4 weeks	Inhibited hepatocellular carcinoma through miR-125b inhibiting regulatory T cell (Treg) accumulation and function.	Li A. M. et al., 2015
	β-glucan	Neutrophils	100 μg/ml	24 h	Induced anti-apoptotic effects on neutrophils relying on activation of Akt-regulated signaling pathways.	Hsu et al., 2002
			10 μg/ml	24 h	Promoted the activation and maturation of immature DC.	Lin et al., 2005
		THP-1, U937 cells	100 μg/ml	72 h	Induced selected monocytic leukemic cell differentiation into DCs with immuno-stimulatory function.	Chan et al., 2007
	A fucose-containing glycoprotein	Con A-stimulated mouse spleen cells	0.01–0.1 μg/ml	72 h	Stimulated the expression of cytokines, especially IL-1, IL-2, and INF-g.	Wang et al., 2002
	F3	BALB/c mice spleen cells	100 μg/ml	48 h	Activated the expression of IL-1, IL-6, IL-12, IFN-c, TNF-a, GM-CSF, G-CSF, and M-CSF.	Chen et al., 2004
	L-fucose (FMS)	LLC1 cell, C57BL/6J mice	240 mg/kg	28 days	Induced antibodies against murine Lewis lung carcinoma cells, with increased antibody-mediated cytotoxicity and reduced production of tumor-associated inflammatory mediators.	Liao et al., 2013
	Proteoglycan	Lymphocytes from BALB/c mice spleens	500 μg/ml	72 h	Activated B cells and expressed CD71 and CD25 on the cell surface. Enhanced the expression of protein kinase C α and protein kinase C γ in B cells.	Zhang et al., 2002
	Triterpenes	A549 cell, C57BL/6 mice	120 mg/kg	14 days	Had anti-lung cancer activity *in vitro* and *in vivo* via enhancement of immunomodulation and induction of cell apoptosis.	Feng et al., 2013
	Ganoderic acid Me	YAC-1, LLC cells, C57BL/6 mice	28 mg/kg	20 days	Up-regulated expression of Nuclear Factor-κB after the treatment of GA-Me, which might be involved in the production of IL-2.	Wang G. et al., 2007
		2LL cells, C57BL/6 mice	10 μg/ml	48 h	Induced the apoptosis of competent T cells and increased the proportion of Treg cells	Que et al., 2014
*G. sinensis*	Lipid extract	U937, HepG2 cells	12.8 μg/ml	72 h	Re-establish the antitumor activity of the immunosuppressive tumor-associated macrophages.	Sun et al., 2011a
*G. applanatum*	Polysaccharide	S180 Transplanted Mice	20 mg/kg	10 days	Restored the NK activity and the IL-2 and IFNy production of the spleen cells, which were suppressed by the tumor.	Gao and Yang, 1991
	Exo-biopolymer (EXP)	S180 cell, BALB/c mice.	80 mg/kg	16 days	Inhibited the growth of solid tumor and increased the natural killer (NK) cell activity.	Jeong et al., 2008
	unknown	Breast cancer cells	Unknown	Unknown	Stimulated macrophages in immunosuppressive breast cancer microenvironment.	Javed et al., 2016
*G. tsugae*	mycelium extracts	C3H/HeN mice	50 mg/kg	10 days	Elevated the splenic NK activity and serum IFN titers.	Won et al., 1992
*G. atrum*	Polysaccharide	S180 cell, Kunming mice	100 mg/kg	18 days	Induced anti-tumor activity via the mitochondrial apoptotic pathway related to activation of host immune response.	Li et al., 2011
		CT26 cell, BALB/c mice	200 mg/kg	14 days	Activated macrophages via TLR4-dependent signaling pathways, improved immunity, and inhibited tumor growth.	Zhang et al., 2013
		RAW264.7 cell, C3H/HeN, C3H/HeJ mice	160 μg/ml	48 h	Induced TNF-a secretion through TLR4/ROS/PI3K/Akt/MAPKs/NF-κB pathways during macrophage activation.	Yu et al., 2014
		CT26 cell, BALB/c mice	200 mg/kg	15 days	Exerted antitumor activity *in vivo* by inducing apoptosis via mitochondria-mediated apoptotic pathway and enhanced host immune system function.	Zhang et al., 2014
			100 mg/kg	18 days	Activated peritoneal macrophages and spleen lymphocytes in cyclophosphamide-treated mice.	Yu et al., 2015a
*G. formosanum*	PS-F2	S180, B16, C26 cells C57BL/6, BALB/c mice	50 mg/kg	24 days	Activated host immune responses against ongoing tumor growth.	Wang et al., 2014

#### Lung cancer

Feng et al. evaluated the inhibitory effect of triterpenes of *G. lucidum* on cell proliferation and tumor growth. The IC_50_ of triterpenes on A549 cells was 24.63 μg/mL (Feng et al., 2013). Triterpenes could significantly inhibit tumor growth in Lewis tumor-bearing mice (30, 60, and 120 mg/kg), and the indices of immune organs, including the spleen and thymus, were increased remarkably by the treatment with triterpenes. Moreover, an *in vitro* study by Liao et al. found that the L-fucose (Fuc)-enriched Reishi polysaccharide fraction (FMS) could inhibit the growth of cancer cells through an increase in the antibody-mediated cytotoxicity and the reduction of the production of tumor-associated inflammatory mediators, particularly monocyte chemoattractant protein-1 (MCP-1). *In vivo* studies showed a significant increase in the peritoneal B1 B-cell population, suggesting the FMS-mediated anti-glycan IgM production (Liao et al., 2013). Sun et al. recently showed that the plasma of patients with lung cancer suppressed the proliferation, CD69 expression, and perforin and granzyme B production in lymphocytes upon activation by PHA (Sun et al., 2014). These effects were partially or fully reversed by *G. lucidum* polysaccharides (GLPS). Furthermore, Que et al. suggested that Ganoderic acid Me, a pure lanostane triterpene of *G. lucidum* contributing to the indoleamine 2,3-dioxygenase, helped create a tolerogenic milieu in lung tumors by directly inducing T cell apoptosis, inhibiting CD8+T cell activation, and enhancing Treg-mediated immunosuppression (Que et al., 2014).

#### Liver cancer

Zhang et al. indicated that, in addition to its direct tumoricidal activity, the lipid extract from *G. sinensis* spores could exert an anticancer effect through the stimulation of the activation of human macrophages/monocytes (Zhang et al., 2009). Furthermore, Shen et al. found that the anticancer mycelia of GLPS could be used to disclose the differential expression of miRNA in human hepatocarcinoma cells through the comprehensive investigation of miRNA expression in polysaccharide-treated cancer cells (Shen et al., 2014). Li et al. elaborated that GLPS significantly suppressed the tumor growth in hepatoma-bearing mice. This effect was associated with an increase in the ratio of the effector T cells (Teffs) to regulatory T cells (Tregs) (Li A. M. et al., 2015). Moreover, GLPS eliminated the Treg-induced suppression Teff proliferation through increased IL-2 secretion.

#### Melanoma

Sun et al. found that GLPS promoted B16F10 melanoma cells to induce lymphocyte proliferation, CD69 and FasL expression, and IFN-γ production. The authors also indicated that GLPS improved the ability of B16F10 cells to activate lymphocytes (Sun et al., 2011b). In addition, the culture supernatant of B16F10 melanoma cells (B16F10-CS) inhibited lymphocyte proliferation and the production of perforin and granzyme B in lymphocytes after induction with phytohemagglutinin and lymphocyte proliferation in the mixed lymphocyte reaction (Sun et al., 2011a). They also found that GLPS could enhance the activity of major histocompatibility complex (MHC) class I molecules and costimulatory molecules, and improve the efficiency of immune cell-mediated cytotoxicity against B16F10 cells (Sun et al., 2012). Barbieri et al. demonstrated that the ethanolic extracts of *G. lucidum* significantly inhibited the release of IL-8, IL-6, MMP-2, and MMP-9 in cancer cells under pro-inflammatory conditions (Barbieri et al., 2017). Wang et al. revealed that the continuous administration of the *G. formosanum* polysaccharide PS-F2 activated the host immune responses against ongoing tumor growth (Wang et al., 2011, 2014).

#### Leukemia

Wang et al. revealed that GLPS might play an indirect role in potentiating antitumor immunity *in vivo* through an increase in the levels of IL-1 and IL-6 (Wang et al., 1997). Lin et al. showed that GLPS promoted the cytotoxicity of specific cytotoxic T-lymphocytes (CTL) induced by dendritic cells (DCs) (Cao and Lin, 2003). These lymphocytes were pulsed with P815 tumor antigens during the stage of antigen presentation and the reported mechanisms of cytotoxicity involved the IFNγ and granzyme B pathways. In addition, the found that GLPS (400 or 100 mg/mL), which promoted CIK cell proliferation and cytotoxicity, enhanced IL-2 and TNF production, and the protein and mRNA expression of granzyme B and perforin in CIK cells through a synergistic interaction with cytokines, decreasing doses of IL-2 and anti-CD3 by 75 and 50%, respectively, which might be irrelevant to nitric oxide (NO) (Zhu and Lin, 2006). Moreover, Chan et al. suggested that GLPS could induce selected monocytic leukemic cell differentiation into DCs with immunostimulatory function (Chan et al., 2007). Chang et al. prepared a water extract of *G. lucidum* and examined its effect on natural killer (NK) cells; they observed that the treatment increased cytotoxicity in NK cells through the stimulation of perforin and granulysin secretion (Chang et al., 2014).

#### Colon cancer

Zhang et al. found that *G. atrum* polysaccharides could activate macrophages via TLR4-dependent signaling pathways, improve immunity, and inhibit tumor growth (Zhang et al., 2013). Wang et al. revealed that the continuous administration of *G. formosanum* polysaccharide PS-F2 activated the host immune responses against ongoing tumor growth (Wang et al., 2014). In addition, Yu et al. indicated that the chemoprotective effects of *G. atrum* polysaccharide might be attributable to its ability to activate peritoneal macrophages and spleen lymphocytes in cyclophosphamide-treated mice (Yu et al., 2015a).

## Major pathways of cancer immunotherapy of ganoderma in immune cells

### Dendritic cells and T-Lymphocytes

**Figure 5:**
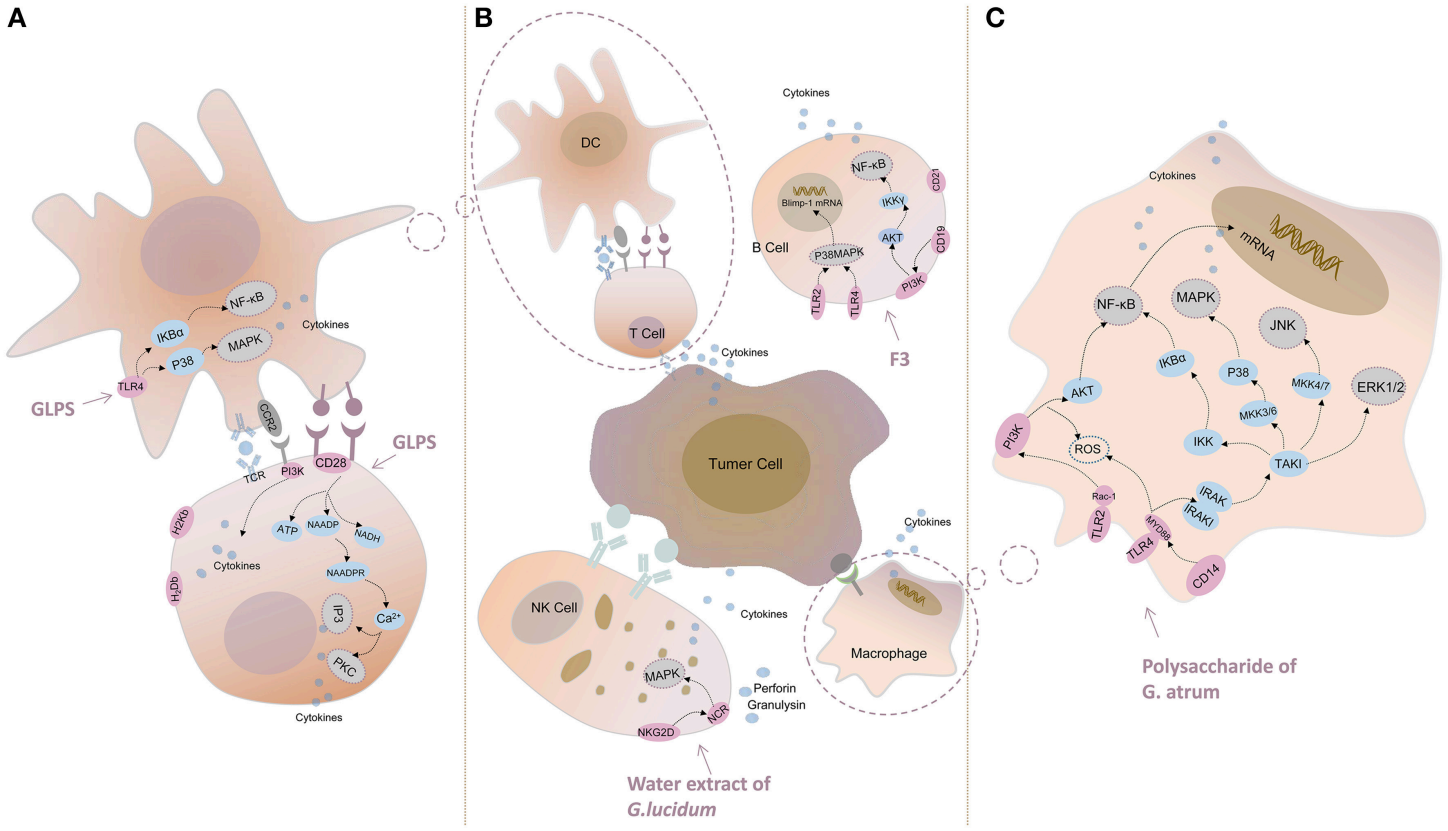
Major pathways of cancer immunotherapy of *Ganoderma* in immune cells. **(A)** GLPS induces NF-κB activation and p38 mitogen-activated protein kinase (MAPK) phosphorylation in DC. GLPS might activate T cells via inositol triphosphate/Ca^2+^ (IP3/Ca^2+^) and protein kinase C (PKC) pathways. **(B)** F3 induces the expression of Blimp-1mRNA through p38 MAPK pathway and mediates intracellular signal through NF-κB pathway in B cell. The water extract of G. lucidum activates NK cells by the mechanism of activating NKG2D/NCR receptors and MAPK signaling pathway. **(C)** The polysaccharide of G. atrum induced macrophage activation through MAPK (JNK, ERK1/2) and NF-κB signaling pathways.

Toll-like receptor (TLR)-4 inhibited the GLPS-induced production of IL-12 and IL-10, which suggested a vital role in DC signaling after incubation with GLPS. Further studies showed that GLPS could augment the activity of κB (IκB) kinase and nuclear factor (NF)-κB inhibitors, as well as the phosphorylation of IκBα and p38 mitogen-activated protein kinase (MAPK) (Lin et al., 2005; Figure 5A).

Sun et al. revealed that GLPS enhanced the effect of H-2K^b^ and H-2D^b^, and B7-1 and B7-2 (two prominent MHC class I molecules in C57BL mice) on B16F10 cells and that the mRNAs of these molecules improved the efficiency of the antitumor cytotoxicity in GLPS-treated cells (Sun et al., 2012). Li et al. inferred that GLPS might activate T cells via the inositol triphosphate/Ca^2+^ (IP3/Ca^2+^) and protein kinase C (PKC) pathways, because the extracellular receptor was bound by GLPS (Li et al., 2013; Li X. L. et al., 2015; Figure 5A).

### B lymphocytes and natural killer cells

Lin et al. showed that the interaction of F3 (the main polysaccharide fraction of G. lucidum) with TLR4/TLR2, followed by signaling through p38 MAPK, was involved in the induction of Blimp-1 mRNA (Figure 5B) and that the intracellular signal was mediated by the NF-κB pathway (Lin et al., 2006).

Chang et al. indicated that G. lucidum induced cytotoxicity in various cancer cell lines through the activation of the NKG2D/NCR receptors and MAPK signaling pathways, which ultimately culminated in the exocytosis of perforin and granulysin (Chang et al., 2014; Figure 5B).

### Macrophage

Kuo et al. revealed that the dried mycelia of *G. lucidum* also induced NF-κB activation in murine RAW264.7 macrophages, which indicated that NF-κB activation was one of the most important signaling pathways (Kuo et al., 2006). Pro-inflammatory cytokines (TNF-α, IL-1β, or IFN-γ) were able to bind to their respective receptors and induce iNOS expression via the activation of NF-κB. Yu et al. indicated that the signaling mechanism might be that of *G. atrum* polysaccharide-induced macrophage activation through TLR4-mediated NF-κB and MAPK (p38, ERK1/2, and JNK) signaling pathways, thereby initiating the release of cytokines, such as TNF-α and IL-1β, and effector molecules, such as NO, in macrophages (Yu et al., 2015b). The results suggested that the polysaccharide of *G. atrum* exerted its antitumor activity through the improvement of immune system functions and acted as an antitumor agent with immunomodulatory activity (Figure 5C). Yu et al. concluded that the polysaccharide of *G. atrum* induced TNF-α secretion through the TLR4/ROS/PI3K/Akt/MAPKs/NF-κB pathways during macrophage activation (Yu et al., 2014). To investigate the possible signaling pathways involved in the activation of macrophages of S180 tumor-bearing mice by the polysaccharide of *G. atrum*, Huang et al. simulated macrophages and observed an increase in the phosphorylation of NF-κB, Akt, and MAPK family proteins, which was indicative of the activation of the NF-κB pathway (Huang et al., 2016). These findings further indicated the possible involvement of the NF-κB signaling pathway in TNF-α secretion and mRNA expression (Figure 5C).

## Clinical studies

Selections of clinical studies are presented. In 2003, Gao et al. investigated the effects of Ganopoly on the immune function of 34 patients with advanced-stage cancer. They found that it enhanced the immune responses in patients with advanced-stage cancer through an increase in the number of CD3^+^ (and similar) cells (Gao et al., 2003). In 2008, Shing et al. found that a 6 months treatment *G. lucidum* increased the mitogen-induced lympho-proliferative responses in immune-compromised children with tumors (Shing et al., 2008). In 2012, a pilot study suggested that the spore powder of *G. lucidum* had beneficial effects on cancer-related fatigue and quality of life in 48 patients with breast cancer undergoing endocrine therapy, without any significant adverse effects. The experimental group made statistically significant improvements in the domains of physical well-being and the fatigue subscale after intervention (Zhao et al., 2012). In addition, a study of five patients with gynecological cancer showed that they achieved stability in the disease after the ingestion of Lingzhi in the form of fruit body water extract and spores (Suprasert et al., 2014). Some modest benefit was also found when the mushroom was administered with standard chemotherapy (Chen and Alpert, 2016).

## Toxicology

The toxicology and immunology of *Ganoderma* have been partly investigated in current studies. Wanmuang et al. presented a case in which fatal fulminant hepatitis occurred after taking Lingzhi powder for 1–2 months (Wanmuang et al., 2007). In addition, a patient was diagnosed with non-Hodgkins lymphoma and presented with chronic watery diarrhea whilst taking Lingzhi (Suprasert et al., 2014). However, no abnormal clinical-symptoms or deaths and no significant difference in body weight and food intake rate was found in Wistar rats during the 30 days administration period (Cheng et al., 2008). No mutagenicity was observed, as indicated by negative results from the Ames test, micronucleus test of polychromatic erythrocyte, sperm abnormality test, and chromosome aberration test in Kunming mice (Zhang et al., 2016).

## Disscusion

The present review provides the most up-to-date analysis of *Ganoderma* research over a 30-years period by using CiteSpaceV and RAW Graphs. We found that the number of studies have increased significantly over time, especially during Stage 3 (Figure 1). We inferred that chemical drugs may exhibit certain side effects. Hence, the medicinal capabilities of the *Ganoderma* fungi have been gradually elucidated. In addition, China, the United States, Malaysia, Japan, and South Korea are the world leaders in *Ganoderma* research, based on outputs and close cooperation among the 84 countries active in the research area (Figure 2). Remarkably, the output of China is ~20% of the total output, which gives it the highest output of these countries. Based on a large amount of data, we summarized the subject categories of the research and found that “Pharmacology & Pharmacy” is the leading category. In the subcategories within pharmacology, immunomodulatory effects and cancer treatment occupy the largest proportion of the eight areas of pharmacology in Chinese-language and English-language articles. These finding revealed a new trend, which was the use of *Ganoderma* in cancer immunotherapy research.

Cancer is a disease with a high death rate. Chemotherapy does not completely meet the needs for cancer treatment and immunotherapy is a promising alternative method owing to the fewer side effects observed. *Ganoderma*, a medicinal mushroom, could be administered as an adjunct to conventional treatment to enhance the tumor response and stimulate host immunity. At the species level, studies on *G. lucidum* predominate; other species are less well-studied. With regard to the effective components, FIPs and polysaccharides are dominant; of which Lz-8 and polysaccharides from *G. lucidum* are the most researched. *Ganoderma* also plays important roles in many aspects of immune regulation for cancer treatment, not only the activation of T or B lymphocytes, macrophages, NK cells, and other immune cells, but in the promotion of the *in vitro* proliferation of undifferentiated spleen cells, and the production of cytokines and antibodies. NF-κB and MAPK, the most comprehensively investigated major pathways, are shown to be activated and release cytokines that subsequently inhibit the growth of tumor cells. TLR-4 is an effective receptor involved in the host defense mechanism of the immune response to polysaccharides. In addition, some researchers have used *Ganoderma* in combination with drug treatments for cancer, such as the combination of GMI and cisplatin and the combination of *G. atrum* polysaccharides with cyclophosphamide to reduce the side effects of the drug. We found that the immunotherapy of lung cancer, liver cancer, melanoma, leukemia, and colon cancer were thoroughly studied *in vivo* and *vitro*, particularly lung and liver cancers. This observation was basically consistent with the channel tropism of *Ganoderma* in TCM theory. Moreover, this review has made a preliminary analysis of the safety of *Ganoderma* through the exploration of the reported toxicology. With regard to adverse effects, there were generally no serious side effects from the use of Lingzhi, but patients should be monitored while receiving Lingzhi, as liver toxicity and chronic watery diarrhea are reported side effects.

*Ganoderma* is one of the most widely used herbal fungi and is a promising anticancer immunotherapy agent owing to its low toxicology and efficacy as a combination therapy. However, the mechanistic pathways lack specificity and do not accurately select specific targets; in addition, most results are derived from *in vitro* studies. Future studies should focus on the combination therapies of *Ganoderma* and clinical chemotherapy drugs to alleviate the side effects of these drugs. Furthermore, the safety and toxicity should be thoroughly explored. The major bioactive components should to be investigated and corresponding *in vivo* pharmacokinetic studies should be performed. The mechanisms underlying immune modulation and interactions should be determined.

## Author contributions

YC conducted and designed the review and wrote the MS. XX and SL contributed to the language editing. LH and JG conducted the designed the review.

### Conflict of interest statement

The authors declare that the research was conducted in the absence of any commercial or financial relationships that could be construed as a potential conflict of interest.
